# Pump Polarization and Size Effects on the Performance of Polymer Lasers

**DOI:** 10.3390/polym11122031

**Published:** 2019-12-07

**Authors:** Chao Chen, Liang Han, Junhua Tong, Xiao Zhang, Shuai Zhang, Tianrui Zhai

**Affiliations:** Institute of Information Photonics Technology and College of Applied Sciences, Beijing University of Technology, Beijing 100124, China; s201706083@emails.bjut.edu.cn (C.C.); hanliang@emails.bjut.edu.cn (L.H.); jhtong@emails.bjut.edu.cn (J.T.); S201806083@emails.bjut.edu.cn (X.Z.); zhangshuai@emails.bjut.edu.cn (S.Z.)

**Keywords:** polymer lasers, pump sizes, pump polarizations

## Abstract

The parameters of a pump have a marked influence on the performance of distributed feedback polymer lasers. Our polymer laser consisted of a grating and a polymer film. We fabricated the grating using interference lithography. The polymer film was spin coated on the grating. A half-wave plate was used to change the pump polarization, and an x-y slit was used to change the pump size. The direction of grating lines were parallel to the x axis of the slit. The laser performance was modified by changing the polarizations and sizes of the pump beam. The lasing threshold increased more rapidly with decreasing pump size in the y direction than in the x direction. The influence of the pump polarization on the lasing threshold for decreasing pump size in the x direction was greater than that for decreasing pump size in the y direction. These results may be useful for the miniaturization of distributed feedback polymer lasers.

## 1. Introduction

Polymer materials can be used in laser devices which have attracted much attention because of their high luminous efficiency, broad photoluminescence (PL) spectra, and high film quality [1,2,3,4,5]. Distributed feedback (DFB) polymer lasers have been intensively investigated due to the small size, low cost, and easy fabrication [6,7,8,9,10]. There are many methods to fabricate polymer lasers, such as interference lithography [11,12], nanoimprinting [13,14], interference ablation [15,16], and electron beam lithography [17,18]. Generally, the threshold, as one of the most important parameters of the polymer lasers, is decided by the balance between the gain and loss. Specifically, the threshold was controlled by the main parameters of the cavity, such as the material [19,20,21,22,23], the type [24,25,26], the quality [27,28], and the size [29,30,31]. Recently, the dependence of the laser threshold on the pump spot diameter was studied in the DFB cavity [29,30]. The effects of the pump polarization on the laser performance has also been investigated [32,33,34]. The role of pump polarization on amplified spontaneous emission and stimulated emission has been studied systematically [32]. Moreover, both the pump polarization and the resonator geometry can be used to tailor the polarization of the output [33,34]. However, the influence of the size of the pump spot on the laser performance is not well understood for the DFB polymer lasers. The combined effects of the pump polarization and size on the laser performance are has not so far been clarified. Actually, the pump polarization and size effects on the performance of DFB polymer lasers can be studied by changing both the pump polarizations and sizes.

In this paper, we studied the influence of the pump polarizations and sizes on the laser performance. The DFB polymer lasers were fabricated by combining interference lithography and spin coating. A half-wave plate was placed in front of the sample to change the polarization of the pump beam. An x-y slit was used to change the size of the pump spot on the sample. The x axis of the slit was parallel to the direction of the grating lines. So, the length and the number of the grating lines in the excitation area are controlled by the x axis and y axis of the slit, respectively. We found that the output intensity decreased and the lasing threshold increased with the pump polarization rotating from the x axis to the y axis of the slit. The lasing threshold decreased more rapidly when decreasing the number of the grating lines than when decreasing the length of the grating lines.

## 2. Materials and Methods

### 2.1. Materials

In our experiment, a diode-pumped solid-state laser (FLARE NX, Coherent, Santa Clara, CA, USA) with 343 nm, 1 ns and 200 Hz was used as an ultraviolet source. The laser was split into two equal beams with each having a power of 5 mW to generate an interference pattern. The photoresist (PR, AR-P3170, Strausberg, Germany) was spin-coated on a glass substrate (15 × 15 × 1 mm) at a speed of 2500 rpm for 30 s and heated for 60 s. Then a PR grating was fabricated by interference lithography with an exposure time of 25 s and a development time of 9 s. The sample was immersed in the developer for 9 s and then in the deionized water for 60 s, forming a grating structure. A conjugated polymer poly ((9,9-dioctylfluorenyl-2,7-diyl)-alt-co-(1,4-benzo-(2,10,3)-thiadiazole)) (F8BT, Sigma-Aldrich, St. Louis, MO, USA) was employed as the gain material. The polymer was dissolved into xylene with a concentration of 23.5 mg/mL. The solution of F8BT was spin-coated onto the grating structure at the speed of 1800 rpm for 30 s, forming a 120 nm polymer film.

### 2.2. Methods

A 200-fs laser with a repetition rate of 1 kHz and a wavelength of 400 nm (coherent) was used as the pump source. The intensity of the laser beam was tuned continuously by a variable optical attenuator. The emission spectra were collected by a spectrometer (Maya 2000 Pro, Ocean Optics, FL, USA). The resolution of the spectrometer was 0.3 nm. The size of the excitation was precisely controlled by an x-y slit.

The simulation was done using the finite element method with the commercial software COMSOL, Gsolver, and EastFDTD. For COMSOL, the simulation was done for solving the eigenmodes (using Radio Frequency(RF)Module/In-Plane Waves/Hybrid-Mode Waves/Eigenfrequency analysis, and the solver was chosen as “Eigenfrequency”).

### 2.3. Fabrication of DFB Polymer Lasers

**Figure 1:**
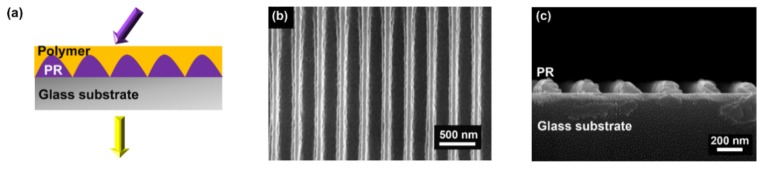
(**a**) Schematic of a distributed feedback (DFB) polymer laser. The purple and the yellow arrows represent the pump and the emission, respectively. (**b**) Top-view SEM image of the grating structure. (**c**) Cross-section view SEM image of the cross section of the grating. Photoresist (PR).

We employed spin-coating and interference lithography to fabricate the laser device. Figure 1a presents a schematic diagram of a DFB polymer laser. Figure 1b,c shows the top-view and cross-section scanning electron microscopy (SEM) images of the grating structure without the polymer film, respectively. The period and the depth of the grating were 340 nm and 120 nm, respectively, as shown in Figure 1b,c. The solution of polymer was spin-coated onto the grating structure, forming a polymer laser.

### 2.4. Characterization

**Figure 2:**
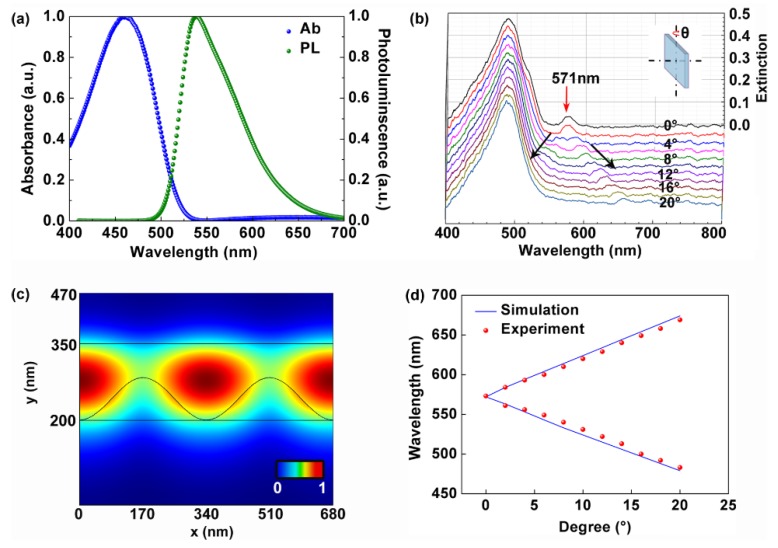
(**a**) The absorption and the photoluminescence (PL) spectra of the polymer film. (**b**) Angle-resolved tuning properties of the waveguide modes of the cavity. (**c**) Electric field distribution of the 571 nm mode of the cavity. (**d**) Simulated spectral shift of the waveguide mode.

Figure 2a shows the spectroscopic properties of the polymer film. In our experiment, a tungsten halogen lamp (HL-2000) was employed as a white light source. The absorption spectrum centered at 460 nm and the PL spectrum peaked at 540 nm. Figure 2b demonstrates the angle-resolved tuning properties of the waveguide modes of the cavity. Under the normal incidence of the white light beam as shown in the inset in Figure 2b (θ = 0), two peaks were observed at 460 nm and 571 nm, respectively. The broad peak at 460 nm was attributed to the absorption of the polymer. The narrow peak at 571 nm corresponded to the waveguide mode of the polymer film. Under the oblique incidence condition, the 571 nm peak split into two branches as indicated by the black arrows in Figure 2b. The angle-resolve tuning rate was about 5 nm per degree for both branches. Figure 2c presents the electric field distribution of the 571 nm mode by using the COMSOL software. It can be clearly observed that the waveguide modes dominated in the polymer film. The spectral shift of the 571 nm mode is simulated in Figure 2d. The tuning rate of the wavelength of the waveguide mode was 5 nm per degree, which agrees well with the experimental results in Figure 2b. All parameters for the simulation were consistent with the structural parameters in Figure 1. The effective refractive indices of polymer, PR, and glass at 571 nm were 1.94, 1.72, and 1.51, respectively. The effective refractive indices were measured by an ellipsometer (ESNano, Ellitop, Beijing, China).

**Figure 3:**
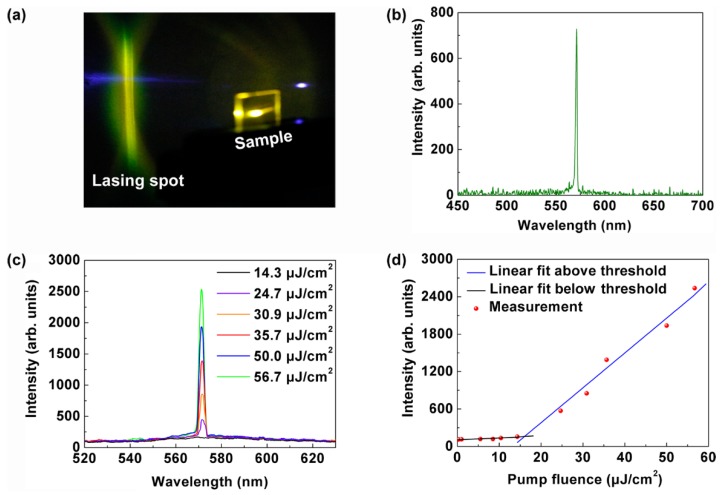
(**a**) Photograph of the operating DFB polymer laser. (**b**) Measured emission spectrum of the DFB polymer laser. (**c**) Measured emission spectra of the polymer laser. (**d**) Evolution of the output intensity as the pump fluence, indicating a threshold of 15 μJ/cm^2^.

Figure 3a shows a photograph of the operating polymer laser. The energy of the pump beam was controlled by an attenuator. The output of the polymer laser was measured by a spectrometer. Figure 3b shows the emission spectrum of the laser device. The emission wavelength was at 571 nm, which was consistent with the peak of the waveguide mode in Figure 2b. This indicated that the laser oscillation was supported by the waveguide. The full width at half maximum (FWHM) of the lasing peak was less than 1 nm above the pump threshold. The output wavelength was not centered on the maximum of PL. The mechanism has been investigated systematically [35]. Figure 3c shows a typical emission spectra of the polymer laser. Figure 3d presents the evolution of the output intensity as the pump fluence, indicating a threshold of 15 μJ/cm^2^. All the thresholds indicated by the dots in our experiment were measured by the method in Figure 3d.

## 3. Results and Discussion

**Figure 4:**
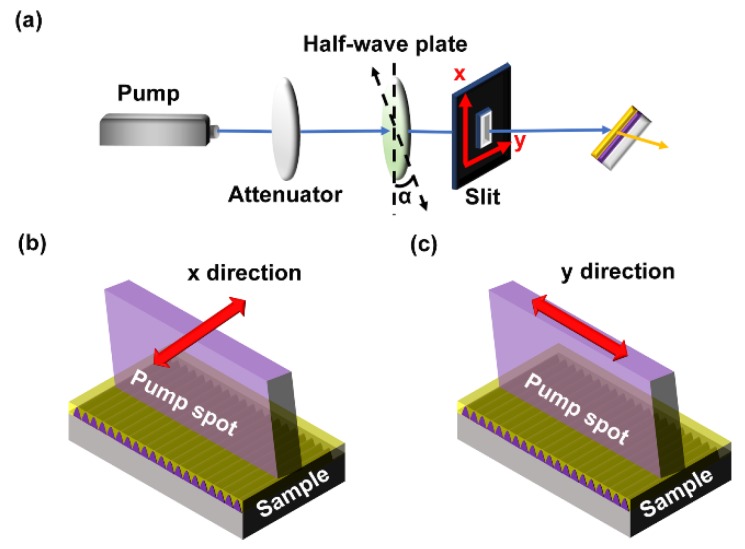
(**a**) Schematic of the optical setup for measuring the pump polarization and size effects of DFB polymer lasers. The size of the pump spot was adjusted by the x-y slit along (**b**) the x direction and (**c**) the y direction.

Figure 4 shows the schematic of the optical setup for investigating the relationship between the laser performance and the polarization and size of the pump beam. A half-wave plate was placed in the optical setup to change the polarization of the pump beam, as shown in Figure 4a. α was the angle between the direction of grating lines and the polarization direction of the pump beam. An x-y slit was used in the optical setup to control the size of the pump spot along the x-y direction. Figure 4b shows that the size of the pump spot was adjusted by the slit along the x direction. So, the length of the grating lines in the excitation area decreased with decreasing the slit width along the x direction. Figure 4c presents that the size of the pump spot was changed by the slit along the y direction. The number of the grating lines in the excitation area decreased with decreasing the slit along the y direction.

**Figure 5:**
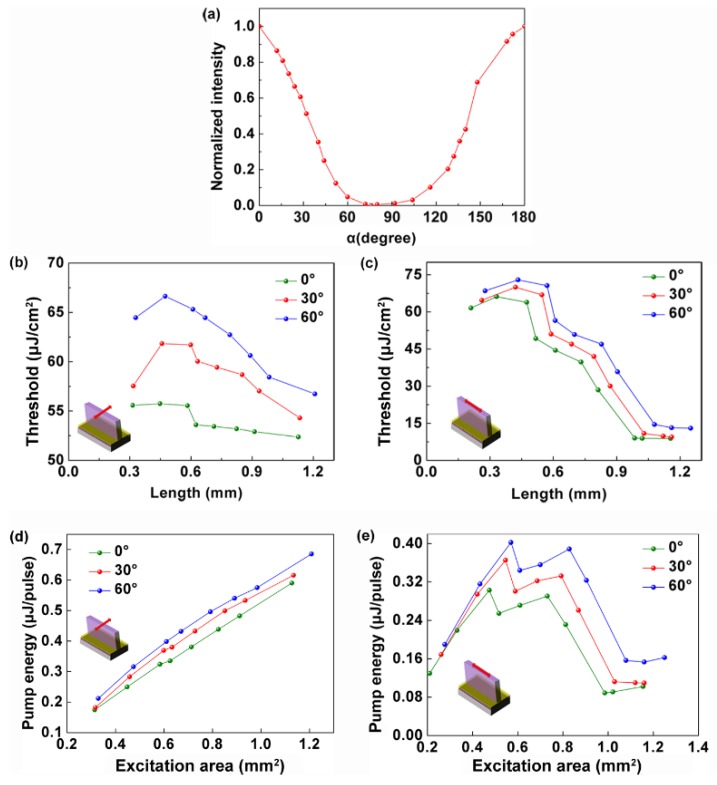
Pump polarization and size effects of DFB polymer lasers. (**a**) Output intensity as a function of the polarization of the pump. The relationship between the lasing threshold and the pump size changed along (**b**) the x direction and (**c**) the y direction with different pump polarizations. The relationship between the pump energy and the pump size along (**d**) the x direction and (**e**) the y direction with different pump polarizations.

Figure 5a presents the relationship between the output intensity and the polarization of the pump beam. Note that the output intensity reached its maximum when the pump polarization was parallel to the grating lines (α = 0). When the polarization direction of the pump beam was perpendicular to the grating line, the output intensity decreased to 0. So, the output intensity was sensitive to the polarization of the pump beam.

Figure 5b,c shows the relationship between the lasing threshold and the size of the pump spot with different pump polarizations. With increasing length of the grating lines in the excitation area in Figure 4b, the lasing threshold decreased slowly as shown in Figure 5b. The decrease rate was 1.1 μJ/cm^2^, 0.8 μJ/cm^2^, and 0.4 μJ/cm^2^ per 100 μm for the 0, 30, and 60 degrees polarization of the pump beam (α), respectively. The polarization direction of the pump must be the same as the direction of the grating lines to obtain low threshold lasing [36]. With increasing the number of the grating lines in the excitation area in Figure 4c, the lasing threshold decreased rapidly as shown in Figure 5c. The decrease rate was 6.6 μJ/cm^2^ per 100 μm, which was almost same for different pump polarizations. This implies that the influence of the pump polarization on the lasing threshold for decreasing pump size in the x direction was greater than that for decreasing pump size in the y direction. Figure 5d,e represents the laser threshold defined by the energy density as a function of the excitation area. The behavior in Figure 5d is clearly linear. So, the laser threshold decreased with reducing the excitation area along the x direction. The behavior of Figure 5e is nonlinear. The behavior is similar to that of Figure 5d below a certain area of about 0.7 mm^2^. Above that, the laser threshold decreased with increasing the excitation area along the y direction.

**Figure 6:**
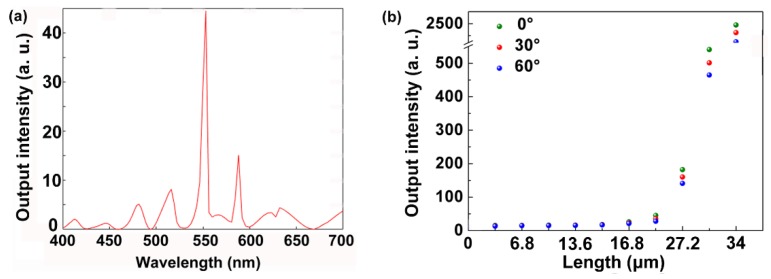
(**a**) Simulated emission spectrum of the DFB laser. (**b**) Simulated output intensity as a function of the pump size along the y direction with different pump polarizations.

Figure 6a demonstrates a simulated emission spectrum of the DFB laser. The lasing wavelength is observed at 571 nm with a FWHM of about 0.7 nm. The electric field distribution of the lasing mode is shown in Figure 2b. All parameters were identical to the structure parameters in Figure 1. Figure 6b presents the relationship between the output intensity and the pump size along the y direction with different pump polarizations. With increasing the pump size along the y direction, the output intensity increased due to the strengthening of the feedback. Note that the output intensity increased significantly when the pump size exceeded a certain length of about 22 μm. With changing the pump polarizations from 0° to 60° at a step of 30°, there was a slight difference among the output intensities. This has been verified by the experiment in Figure 5c.

## 4. Conclusions

The pump polarization and size effects of the DFB polymer laser were studied systematically. The laser device consisted of a grating and a polymer waveguide, which was fabricated by combining interference lithography and the spin-coating method. A half-wave plate and an x-y slit were used to control the pump polarization and the pump size, respectively. The output intensity and the lasing threshold were strongly affected by the pump polarization. The relationship between the lasing threshold and the pump size was also revealed. These results may be helpful in miniaturizing DFB polymer lasers.
